# Unusual case of intraocular medulloepithelioma in an adult male

**DOI:** 10.22336/rjo.2021.61

**Published:** 2021

**Authors:** Obaidur Rehman, Subina Narang, Shifali Nayyar, Phiza Aggarwal

**Affiliations:** * Department of Ophthalmology, Government Medical College and Hospital, Chandigarh, India; ** Department of Pathology, Government Medical College and Hospital, Chandigarh, India

**Keywords:** medulloepithelioma, teratoid tumor, teratoid medulloepithelioma, adult medulloepithelioma

## Abstract

Medulloepithelioma is a rare tumor of the eye, arising from the posterior segment. This embryonic tumor is mostly seen in children and is very rare in adult population.

This case report presents the case of a 39-year-old Indian male, who had gradual vision loss over 4 years in his left eye with new onset of pain. He was referred to our center in view of secondary cataract and intraocular mass. Vision in right eye was 20/ 20 while left eye had no light perception at presentation. Ocular examination of the left eye revealed shallow anterior chamber, florid iris neovascularization, raised intraocular pressure and cataractous lens. B-scan ultrasonography showed a heterogenous mass filling the entire globe. MRI scan confirmed the finding, showing a mass hyper-intense to vitreous. No invasion of optic nerve or sclera was observed. Left eye enucleation with PMMA implant placement was performed and histopathology confirmed the diagnosis of benign teratoid medulloepithelioma. At the time of submission of this report, the patient was still under follow-up and had no detectable metastases at 15 months follow-up.

This report highlights a very rare case of embryonic tumor in adult male, which could be managed successfully with a high index of suspicion and timely intervention.

## Introduction

Medulloepithelioma of the eye is an unusual and rare embryonic tumor, seen mostly in children and with very limited occurrence in adults. Origin of this tumor is noted most from the medullary epithelium of the ciliary body, but occurrence from the retina and optic nerve has also been seen [**1**]. Histopathology is characterized by presence of pseudostratified epithelium arranged as cords or ribbons along with loose connective tissue, appearing much like developing neurosensory retina and vitreous [**2**].

We report a case of a 39-year-old Indian male, presenting with left eye complicated cataract with neovascular glaucoma and ultrasound showing intraocular mass lesion. He was provisionally diagnosed to have retinal medulloepithelioma and managed successfully with enucleation and placement of PMMA implant. Histopathology confirmed the diagnosis. There was no evidence of metastasis at 15 months follow-up.

## Case Report

A 39-year-old Indian male was referred to the retina services of our institute with the diagnosis of left eye secondary cataract with intraocular mass lesion. The patient complained of decreased vision in his left eye for the past 4 years. The loss in vision was insidious in onset and gradually progressed over the past 4 years. Initially, it was painless, but for the past few days the patient started experiencing intermittent pain, which was mild to moderate in intensity. 

On presentation, patient had an unaided vision of 20/ 20 in the right eye and no light perception in the left eye. Ocular adnexa were normal in both eyes and extraocular movements were full, free, and painless in both the eyes.

**Fig. 1:**
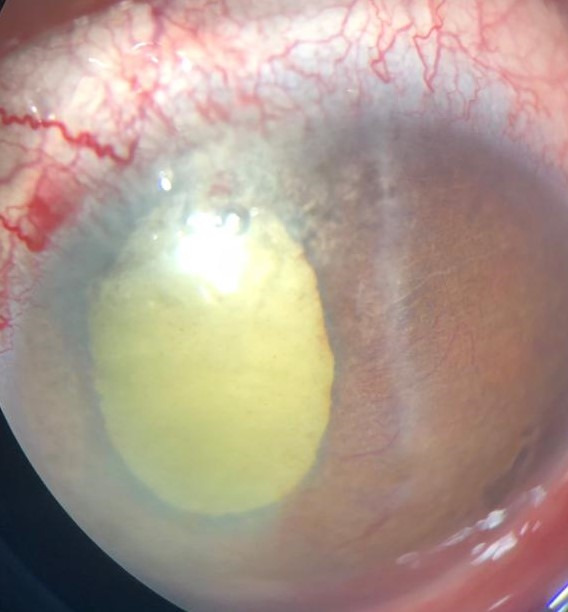
Anterior segment image of left eye showing shallow anterior chamber, florid neovascularization of iris and cataractous lens

Ocular examination of the right eye was essentially normal. Anterior segment examination of the left eye revealed a shallow anterior chamber with presence of anterior synechiae and florid neovascularization of the Iris (**Fig. 1**). Pupil of the left eye was mid-dilated and non-reactive to light. The lens in left eye was cataractous and no view of posterior segment was possible on Indirect Ophthalmoscopy. Intraocular pressures (Goldmann applanation) were 16- and 30-mmHg in right and left eyes, respectively.

**Fig. 2:**
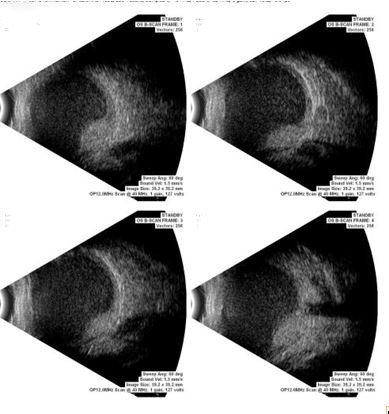
B-scan ultrasonography image showing heterogenous mass in the posterior segment, hypoechoic anteriorly and hyperechoic posteriorly

**Fig. 3:**
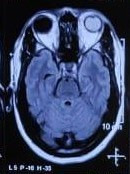
T1W MRI scan showing mass filling the entire left eye

B-scan ultrasonography of the left eye revealed heterogenous mass lesion occupying the entire globe (**Fig. 2**). The lesion was primarily hypoechoic anteriorly and showed a small hyperechoic area posteriorly. Axial T1 weighted - MR image revealed mass lesion filling the entire left globe, which was hyper-intense to vitreous (**Fig. 3**). No invasion of the optic nerve or sclera was noted.

Systemic examination and metastatic work up of the patient, including blood tests and imaging, ruled out the presence of any metastases.

Based on the clinical presentation and radiological findings, differential diagnoses of choroidal melanoma and intraocular medulloepithelioma were suspected. In view of the blind eye, the patient was planned for left eye enucleation in general anaesthesia.

Under General Anaesthesia, left eye enucleation using myo-conjunctival technique was done, with the removal of 17mm of the optic nerve and a PMMA implant size 20 was inserted. Tenon’s capsule was sutured over the implant and finally the conjunctiva was sutured. A conformer was placed over the sutured conjunctiva. The enucleated eyeball was sent for histopathological examination.

**Fig. 4:**
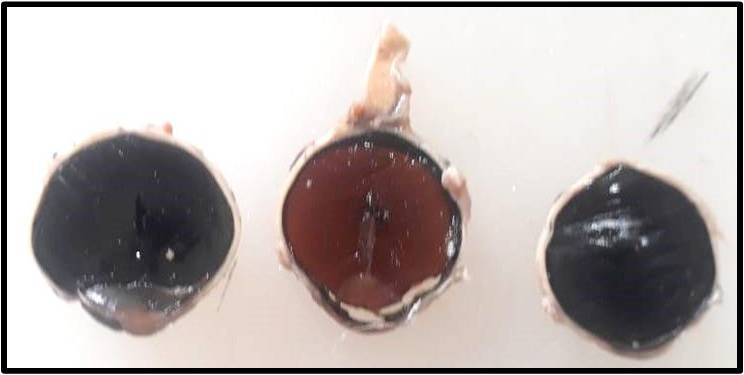
Gross pathology specimen showing tan-colored mass occupying entire posterior segment

**Fig. 5:**
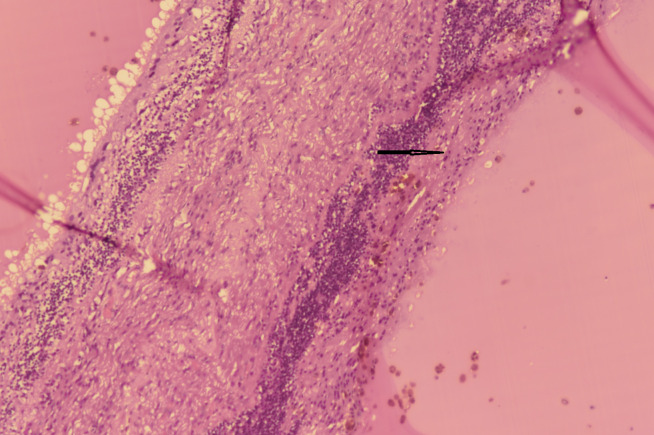
Histopathology showing primitive neuroepithelium in a hypocellular stroma (marked with black arrow)

On gross examination, the histopathological study revealed a grey brown to grey tan mass in the posterior segment, arising from the retina, measuring 1.8 x 1.8 x 1.5 cm, and filling the entire posterior segment (**Fig. 4**). Microscopic examination showed presence of primitive medullary epithelium. Sheets of primitive neuroepithelial cells were seen in a hypocellular stroma (marked with black arrow in **Fig. 5**). Monomorphic tumor cells were seen arranged singly scattered, having salt and pepper chromatin. Histopathology was consistent with that of benign teratoid medulloepithelioma.

Post-operative period was eventful and patient has been on regular follow-up. No metastases have been detected at 15 months follow-up.

## Discussion

Intraocular medulloepithelioma is a rare embryonic tumor, with a reported incidence of 1 per 4,50,000-10,00,000 population [**3**]. It has been seen mainly in children, with very few cases reported in adults. Medulloepithelioma in adults might represent delayed transformation of a pre-existent retinal anlage [**4**].

Floyd and co-workers reported a case of intraocular medulloepithelioma in a 79-year-old patient in 1982, arising from ciliary body and was managed successfully with enucleation [**5**]. Sosinska-Mielcarek and colleagues have also reported a case on intraocular medulloepithelioma in a 44-year-old man in 2006, who was started on chemotherapy but died six months later [**6**]. Origin of medulloepithelioma in the eye is seen most commonly from the ciliary body, but tumors arising from the optic disc, iris, and retinal stalk have also been described [**7**]. 

It is classically unilateral, showing no preference for either eye [**4**]. Initially, patients are asymptomatic and may later present with symptoms such as vision loss, leukocoria, pain, visible intraocular mass, or red eye. Vision loss is due to cataract formation, lens subluxation or neovascular glaucoma. The index case also manifested as neovascular glaucoma [**8**]. The presence of cataract, irregular anterior chamber and neovascular glaucoma raised the suspicion of mass lesion in the present case and radiological investigations containing both solid and cystic areas clinched the diagnosis.

Primary misdiagnosis is reported in 39% of cases [**9**]. Since it is mainly seen in children, it should be differentiated from retinoblastoma, which has characteristic calcifications on B-scan and MRI, but not seen in medulloepithelioma. In adults, differentiation from more common tumors such as choroidal melanoma is required, which shows dome shaped/ mushroom shaped tumor on B-scan, choroidal excavation and subretinal fluid. A high index of suspicion is required to clinch the diagnosis of medulloepithelioma and offer timely management to these cases. Prognosis is generally good and the five-year survival rate is 90-95% if diagnosed early and there is no extraocular spread. However, if not treated in time, deaths have been reported in these cases [**4**].

Enucleation is the standard treatment modality for advanced tumors and neovascular glaucoma. It should be performed with care and least manipulation to avoid spillage of tumor cells [**4**]. Smaller tumors may be treated with cryotherapy, radiotherapy or local resection or plaque brachytherapy [**10**].

## Conclusion

Intraocular medulloepithelioma is very rare in adult population but this case report highlighted that it should be kept in mind as a diagnosis even in adults. A high index of suspicion and timely intervention in these cases is required for a successful long-term outcome.

**Conflict of Interest statement**

Nil.

**Informed Consent and Human and Animal Rights statement**

Any information that could contribute to identify patients, including patients’ names, initials or hospital numbers have not been published in written descriptions or photographs. The manuscript was shown to the patient before submission to the journal and an explicit informed consent was obtained for purpose of publication. 

**Authorization for the use of human subjects**

Ethical approval: The research related to human use complies with all the relevant national regulations, institutional policies, is in accordance with the tenets of the Helsinki Declaration, and has been approved by the review board of Government Medical College and Hospital, Chandigarh, India.

**Acknowledgements**

None.

**Sources of Funding**

None.

**Disclosures**

None.

**Prior publication**

Nil.
